# Retention of Urine, with Treatment

**Published:** 1858-07

**Authors:** Wm. Abram Love

**Affiliations:** Palmyra, Lee County, Georgia


### ARTICLE IV.

*Retention of Urine, with Treatment.* By Wm. Abram Love, M. D., Palmyra, Lee County, Georgia.

In an article copied into the *Southern Medical and Surgical Journal*, for 1850, p. 759—coming indirectly from *L'Union Medicale*, M. Cazenave recommends (*as a new method of relieving retention of urine*), the use of ice and ice water, to be thrown into the rectum and applied to the perineum—the object of which, was to relieve the engorgement of the prostate gland—(the cause of the retention)—and as a stimulus to cause contraction of the bladder.

This treatment, though it may have been *new* with M. Cazenave, and doubtless original, was nevertheless practiced by my preceptor—the late Dr. Samuel George Morton, of Philadelphia—long before the appearance of M. Cazenave's article, and was by him recommended to his private students, while I was in his office in 1845–6. How long it had been his practice, I know not. *He did not present it as new then*, but stated that he had been in *the habit of resorting to this treatment.* He furthermore cautioned his class to be careful in the use of cold in cases where it would be likely to act too powerfully as a sedative, as in the cold stages of Intermittents—in the advanced stages of low forms of fever where the vital forces were very much depressed, &c.

I do not know when M. Cazenave first adopted this treatment, nor do I know when Dr. Morton commenced this practice, but I do know that he spoke of it, and recommended it as *his practice* as early as 1845. I feel that it is but justice to make this statement before giving my own experience in a different mode of affecting the same object, into the discovery of which, the practice of Dr. Morton, accidentally led me.

In the Spring of 1848, I was much annoyed by retention of urine in the case of a little patient—six months old—then suffering from a protracted attack of a low form of fever. The urine had for some time been voided with difficulty—the vital forces were very much reduced—the bowels, though very much disposed to *run off*, had become greatly distended with gas, when complete retention of urine ensued. In the progress of the case, the bladder became very much distended, when the ordinary remedies recommended in such cases were resorted to, but without giving relief. Not being provided with a suitable catheter, I determined to risk the cold injections and cold applications. With this intention, the nozzle of a self-injecting pump was passed into the rectum—it becoming accidentally detached from the pump-tube, leaving the outer extremity free—a quantity of gas escaped from the bowels, and to my gratification, the discharge of gas was followed by a free flow of urine. I returned the tube nozzle to the rectum again the following morning, and repeated it the next evening with a like result; after which time the bladder discharged its contents without artificial aid.

The result of this course led me to conclude that the retention depended upon a triple cause.

1st. Passive congestion of the prostate gland.

2nd. Mechanical pressure upon the same from distention of the rectum and other abdominal viscera; and,

3rd. Partial loss of tone in the bladder itself.

The discharge of the gas removed the mechanical pressure from the enlarged prostate—the contraction of the rectum stimulated the bladder to contraction, which was then sufficient to overcome the resistance of the sphincter vesicœ, and the pressure of the passive engorgement around the urethra.

The last ten years' experience has convinced me of the correctness of these conclusions, and saved me much trouble in cases of protracted illness among children in which retention of urine would otherwise have proved one of the most distressing and troublesome accompaniments.

In one well marked case occurring in the Spring of 1854, my little patient was relieved regularly for more than a week by the mother introducing from time to time, the tube of a common syringe. In this case the repeated use of ice and ice water would have proved fatal, and the re-introduction of the catheter from time to time for so long a period would almost necessarily have resulted in serious injury to the urethra and prostate gland, to say nothing of the trouble and difficulty of performing the operation.

I have always found it necessary to have the lower bowels thoroughly evacuated by enema, and in some cases—where the tone of the bladder is too far spent—the cold douche to arouse it, before it would contract properly.

In cases where the prostatic engorgement is active in its character, this treatment will fail; but in these cases there is no risk in resorting to the ice and ice water injections, as recommended by Dr. Morton and M. Cazenave.

The use of the tube is most effectual in those cases where the vital forces are most depressed—where the peristaltic action of the bowels is very much weakened—where tympanitis exists to the greatest extent, and where the contractions of the rectum are overcome by the sphincter muscles, and there is regurgitation of gas into the colon.

I will mention *en passant*, that I have been persuaded that in cases where tympanitis exists in an inordinate degree, great relief may often be extended to a patient, and much damage to the viscera prevented, by thus providing for the escape of gas, or even by the introduction of a gum elastic catheter into the sigmoid flexure of the colon. It would relieve the inordinate and injurious distention of the bowels, take off the pressure from the diaphragm, and make respiration free and easy. This cannot always be accomplished by the use of absorbents, &c.

That the removal of the gases, as directed will relieve retention of urine in many cases, I am fully convinced from numerous trials during the last ten years. This remedy I have found invaluable, too, from its simplicity. A tube for the purpose—the nozzle of a syringe, a piece of reed, or a large sized quill, in the hands of the mother or nurse—with instructions as to its use, will often save the physician many visits and re-introductions of the catheter, aside from the incalculable benefit, it may prove, or the suffering it may save, to his little patient.

I did not intend to write an article on *Ischuria*, nor do I

now, but simply to comply with your request, and give you the treatment which had in numerous instances proved so satisfactory in my own hands. Should it be the means of affording more speedy relief to any little sufferer, it will have amply requited me for the few moments I have spent in penning these lines. For what it is worth, I give it to you. You have my permission to put it into shape and use it as you like.

